# Overexpression of *BpCUC2* Influences Leaf Shape and Internode Development in *Betula pendula*

**DOI:** 10.3390/ijms20194722

**Published:** 2019-09-23

**Authors:** Chaoyi Liu, Huanwen Xu, Rui Han, Shuo Wang, Guifeng Liu, Su Chen, Jiying Chen, Xiuyan Bian, Jing Jiang

**Affiliations:** State Key Laboratory of Tree Genetics and Breeding, Northeast Forestry University, 26 Hexing Road, Harbin 150040, China; 15245010786@163.com (C.L.); xhwnefu@163.com (H.X.); 2304057514@qq.com (R.H.); 764928481@qq.com (S.W.); liuguifeng@126.com (G.L.); 157872345@qq.com (S.C.); 1182548215@qq.com (J.C.); bianxydblydx@163.com (X.B.)

**Keywords:** *BpCUC2*, *BpmiR164*, leaf, internode, *Betula pendula*

## Abstract

The *CUP-SHAPED COTYLEDON 2* (*CUC2*) gene, which is negatively regulated by *microRNA164* (*miR164*), has been specifically linked to the regulation of leaf margin serration and the maintenance of phyllotaxy in model plants. However, few studies have investigated these effects in woody plants. In this study, we integrated genomic, transcriptomic, and physiology approaches to explore the function of *BpCUC2* gene in *Betula pendula* growth and development. Our results showed that *Betula pendula* plants overexpressing *BpCUC2*, which is targeted by *BpmiR164*, exhibit shortened internodes and abnormal leaf shapes. Subsequent analysis indicated that the short internodes of *BpCUC2* overexpressed transgenic lines and were due to decreased epidermal cell size. Moreover, transcriptome analysis, yeast one-hybrid assays, and ChIP-PCR suggested that BpCUC2 directly binds to the LTRECOREATCOR15 (CCGAC), CAREOSREP1 (CAACTC), and BIHD1OS (TGTCA) motifs of a series of IAA-related and cyclin-related genes to regulate expression. These results may be useful to our understanding of the functional role and genetic regulation of *BpCUC2*.

## 1. Introduction

Plant morphogenesis and organ development are dependent on complex regulatory mechanisms [1]. *Arabidopsis CUP-SHAPED COTYLEDON 2 (CUC2)* gene encoding a NAC-domain transcription factor plays an important role in the development of organ boundaries, internodes, and leaves in plants [2,3]. Double mutants affected in *CUC2* and another redundant genes, including *CUC1*, show near complete fusion of the cotyledons [3]. *CUC* genes also control shoot apical meristem (SAM) formation and cotyledon development through their interactions with *SHOOT MERISTEMLESS* (*STM*) [2]. MicroRNAs (miRNAs) are short non-coding RNAs of ~21 nt in length that act through translational repression and mRNA cleavage of the target gene [4]. The *Arabidopsis miR164* family consists of three members: *miR164a*, *miR164b*, and *miR164c*. They negatively regulate several NAC family transcription factors, including *CUC2* [5,6,7]. Enhanced leaf serration and abnormal phyllotactic patterns were observed following the expression of *miR164*-*resistant CUC2* in *Arabidopsis thaliana*, which indicates that *miR164*-*mediated* repression of *CUC2* is necessary for the correct control of leaf margin serration [8] and phyllotaxy maintenance [9]. Although many studies have assessed the function of *CUC2* in model plants, studies in tree species remain limited.

Birch trees are important broad-leaved fast-growing tree species and one of the most important commercial timber tree species for paper, furniture, and plywood production in China [10]. *Betula pendula* ‘*Dalecarlica*,’ which is an intraspecific variant of *B. pendula*, has great importance in ornamental and classification value [11,12]. In our previous studies, the key genes controlling leaf serrated formation were assessed, and the transcriptomes of *B.pendula* ‘*Dalecarlica*’ and *B.pendula* plants were compared. Among 17,146 uni-genes that were differentially expressed, *BpCUC2* was significantly up-regulated in *B.pendula* ‘*Dalecarlica*’ [10], which indicates its association with leaf margin development.

To investigate roles of *BpCUC2* in *Betula pendula* growth and development, we examined the phenotypes of transgenic lines with additional copy/copies of *BpCUC2*. In addition, several target genes of *BpCUC2* were identified by transcriptome analysis, yeast one-hybrid assays, and ChIP-PCR. We also examined the phenotypes of *BpmiR164* transgenic lines. The data indicate that *BpCUC2* is an important player in the regulation of leaf shape and internode development of birch. *BpmiR164* was identified as a negative regulator of *BpCUC2*, and BpCUC2 primarily acted on a range of IAA-related and cyclin-related genes. 

As an ornamental and timber species, it is of great significance to study the key gene regulating the growth and development of birch. Our results reveal the important role of *BpCUC2* in leaf shape and internode development of *Betula pendula*, and provide a theoretical reference for designed breeding and variety improvements of *Betula pendula*.

## 2. Results

### 2.1. Identification of the CUC2 Gene in Betula pendula

**Figure 1:**
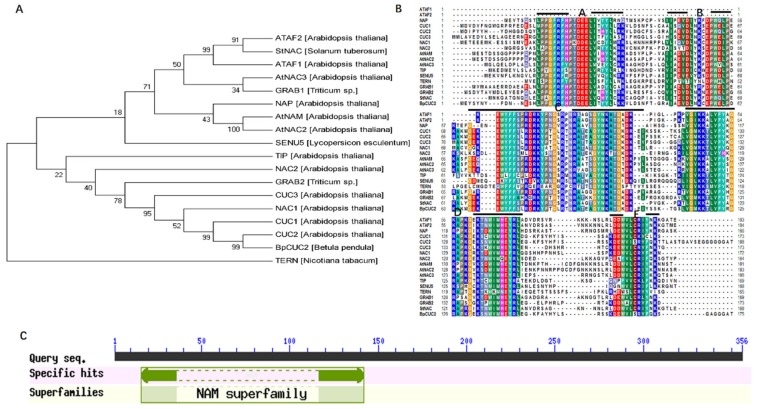
*BpCUC2* sequence analysis. (**A**) Unrooted phylogenetic tree of the NAC domains was depicted by the Mega 5.0 program, and constructed by the neighbor-joining method. Numbers near the branches represent bootstrap values based on 1000 replications. (**B**) ClustalW alignment of BpCUC2 and related NAC family proteins. (**C**) NAM superfamily domain of BpCUC2.

In *Arabidopsis*, *CUC2* plays multiple roles, including embryonic meristem formation, cotyledon separation, and leaf development [13,14,15]. Studies on the function of *CUC2* in birch are lacking. In this scenario, we identified a *CUC2* homolog in *B. pendula*. Unrooted phylogenetic tree of the NAC domains show that BpCUC2 was evolutionarily close to AtCUC2 (Figure 1A). The C-terminal of the BpCUC2 was highly conserved and had typical A, B, C, D, and E domains of the NAC (NAM/ATAF/CUC) family (Figure 1B). In addition, BpCUC2 includes a conserved NAM domain as analyzed by the conserved utility of NCBI, which indicates that BpCUC2 is a member of the NAM superfamily (Figure 1C).

### 2.2. Functional Analysis of BpCUC2 Gene

**Figure 2:**
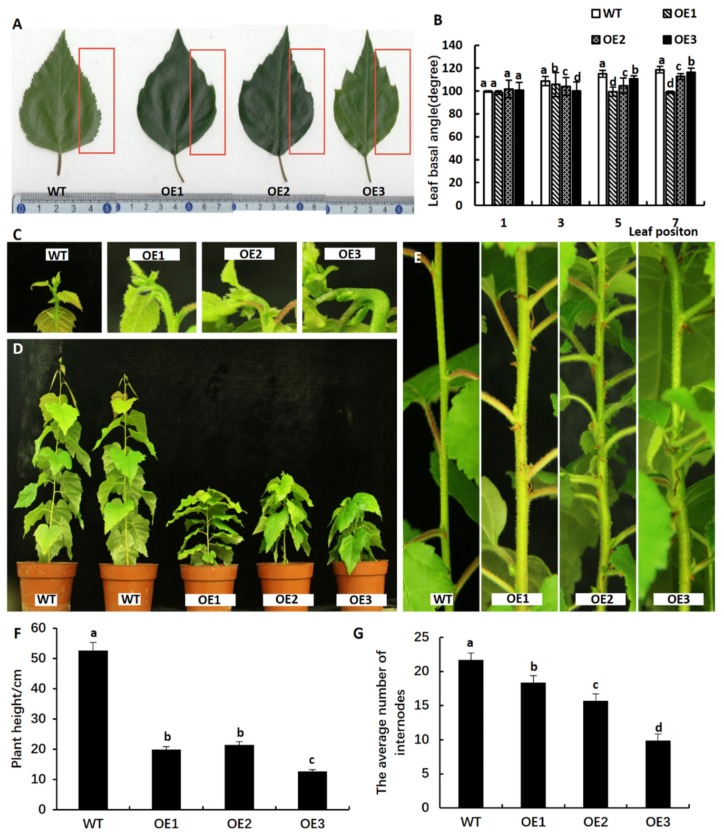
Phenotype of *BpCUC2* OE lines. (**A**) Shape of mature leaves between two-year old *BpCUC2* OE and WT lines. Red boxes show differences in leaf margins among the lines. (**B**) Leaf basal angle of the first, third, fifth, and seventh leaves of two-year old wild type and OE plants. Different letters marked on each column represent significant differences when analyzed by one-way ANOVA and a multiple comparison using Duncan’s test at *P* < 0.05. Error bars represent the standard deviation (SD) of each line. Data indicate means ± SD (*n* = 5). (**C**) Apical bud shape of one-year old *BpCUC2* OE and WT lines. (**D**) Dwarf phenotype of one-year old *BpCUC2* OE in comparison with WT lines. (**E**) Internode of one-year old *BpCUC2* OE lines showing an abnormal phyllotactic pattern. (**F**) Plant height of one-year old *BpCUC2* OE and WT lines. (**G**) Average number of internodes of one-year old *BpCUC2* OE and WT lines. Error bars indicate the SD (Standard Deviation) of each line. Data indicate means ± SD (*n* = 15, *P* < 0.05).

To further explore the biological functions of *BpCUC2*, We overexpressed *BpCUC2* under control of the constitutive CaMV 35S promoter to obtain additional copy/copies of the wild type allele in *B. pendula* by *Agrobacterium*-mediated transformation. We obtained three independent transgenic lines (OE1-OE3) (see Figure S1). We determined the expression of *BpCUC2* in the buds of a seedling stage and one-year-old WT (wild-type) and transgenic lines. Due to the growth characteristics of birch, it grows in Northern China in May and stops growing in September. We, therefore, determined the expression of *BpCUC2* from May to August in WT and transgenic line leaves. Analysis of the expression patterns showed that *BpCUC2* was overexpressed at different developmental stages and in different organs in *BpCUC2* OE lines (see Figure S2). Transgenic birch overexpressing (OE) *BpCUC2* showed multiple phenotypical defects, including abnormal leaves (Figure 2A,B), curved buds, and a severe dwarf phenotype (Figure 2C,D). Mature leaf margin smoothness differed between WT and OE lines, as the number of first and second order leaf teeth were reduced in *BpCUC2* OE lines. In particular, *BpCUC2* OE lines lack third order leaf teeth (see Figure S3A–D). From the first leaf to the eighth leaf, the number of leaf teeth of the wild type line gradually increased with leaf development, while the number of leaf teeth of transgenic lines significantly decreased (see Figure S3A–D), which indicates that, as the leaves develop, many leaf teeth disappeared as they failed to be separated. We also followed early leaf development between WT and the *BpCUC2* OE1 line to further understand the role of *BpCUC2* in the ontogeny of birch leaves. In the OE1 leaves, teeth initiated at the early leaf developmental stage and continued to grow to levels comparable to the wild type (see Figure S4). These results indicate that the smooth margins may not be due to defective teeth initiation or growth during the early stages of leaf development, but because the teeth fail to be separated during late stage developmental stages. Apical buds of *BpCUC2* OE lines were bent at ~90 ° (Figure 2C). In wild type (WT) birch, leaves on the stems were well-organized. However, in *BpCUC2* OE lines, leaves were abnormally distributed along the stems (see Figure 2E and Figure S5). The average height and internode numbers of *BpCUC2* OE lines were significantly reduced (Figure 2F–2G), which indicates that the overexpression of *BpCUC2* affected the growth and development of birch. Because abnormal phyllotactic patterns are often associated with perturbed meristem sizes [16], we analyzed the meristem width of *BpCUC2* OE lines. The widths of *BpCUC2* OE lines were comparable to the wild type (see Figure S6).

**Figure 3:**
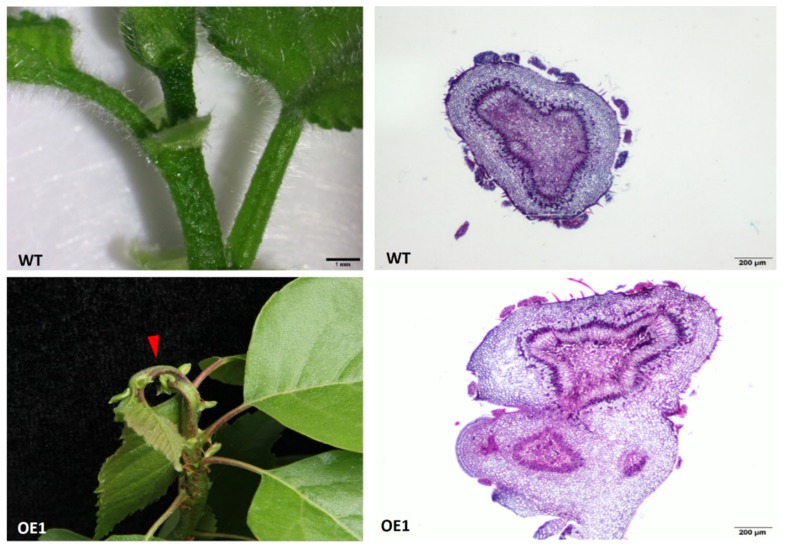
Cross-sections of WT and OE1 stems reveal fused petioles and stems in OE1 (arrowheads). Scale bars are indicated. OE1 represents *BpCUC2* OE1 transgenic lines.

All replicates of the *BpCUC2* OE lines displayed petiole and stem fusion (petiole-stem) during vegetative development. Sectioning analysis revealed the presence of two distinct vascular bundles in the fused petiole-stem (Figure 3). Leaf basal angles of mature leaves from *BpCUC2* OE lines were significantly smaller than those of the wild type (Figure 2B). Leaf margins of the *BpCUC2* OE lines were smoother than those of the wild type (see Figure S3A–D). These data indicate that the ectopic and excessive expression of *BpCUC2* influences the developmental processes of birch.

### 2.3. BpCUC2 Affects Epidermal Cell Size and Cell Number

**Figure 4:**
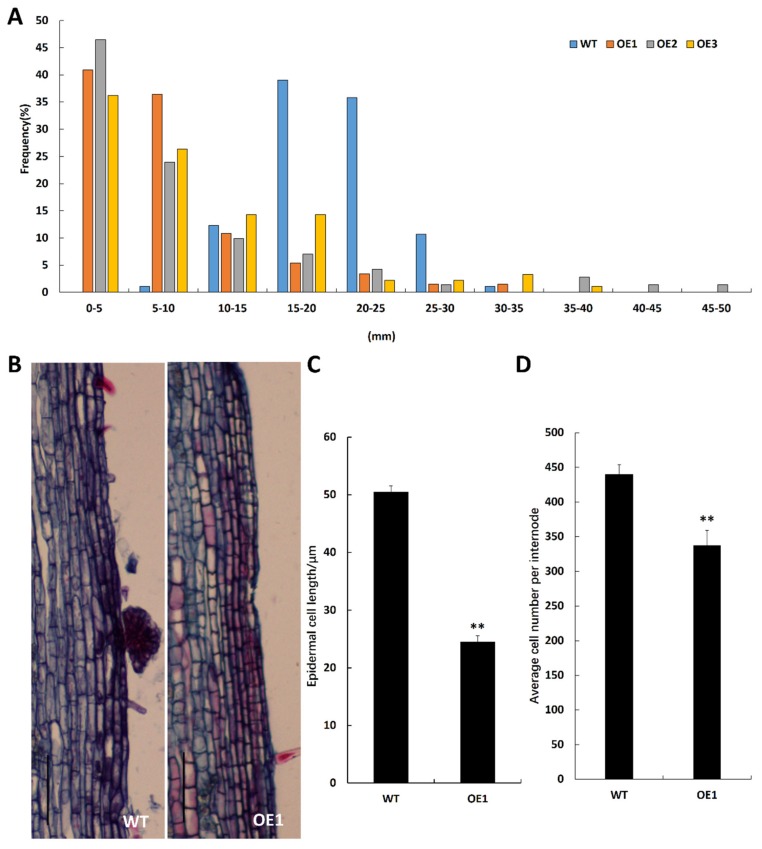
Internode and epidermal cell lengths between WT and *BpCUC2* OE lines. (**A**) Percentage of total internode lengths that fell into 5 mm classes are shown. A total of 15 plants were analyzed for each genotype. (**B**) Longitudinal sections of WT and OE1 stems reveal differential epidermal cell sizes. Scale bar = 100 μM. (**C,D**) Epidermal cell lengths and the cell number of wild type and OE1. Asterisks (**) represent significant differences among the lines based on a Student’s t-test (*n* = 5, *P* < 0.05) and approximately 100 cells were counted per genotype.

Since *BpCUC2* OE lines showed a dwarf phenotype, we measured the internode lengths of WT and *BpCUC2* OE lines. We found that 90% of the internodes in wild type lines were 1.5 cm to 2.5 cm in length. However, 60% of the internodes in *BpCUC2* OE lines were less than 1 cm in length (Figure 4A). We next measured the stem epidermal-cell length and cell number of the wild type and *BpCUC2* OE lines. We found that the epidermal-cell length of OE plants were ~50% of wild type lines (Figure 4B,C) and that the cell number was 76.8% of wild type lines. The shortened internodes of *BpCUC2* OE lines were due to the inhibition in both epidermal cells’ size and cell number.

### 2.4. Identification of BpCUC2 Regulated Genes

**Figure 5:**
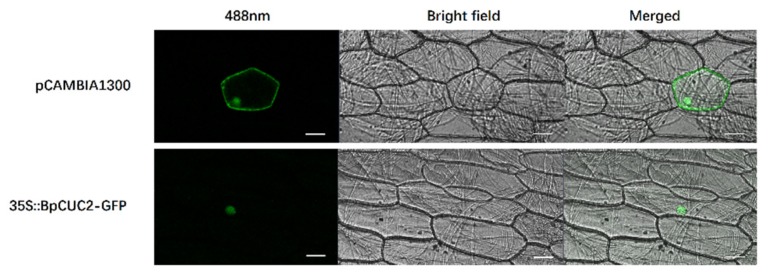
Subcellular localization of BpCUC2. Fluorescence signals show the localization of BpCUC2-GFP in the nuclei of onion epidermal cells. Merged: bright-field and GFP merged images. Scale bar = 50 μM.

*CUC2* was reported as a transcription factor in *Arabidopsis* [2,17]. The *CaMV35S::BpCUC2*-*GFP* plasmid was transfected into onion epidermal cells using particle bombardment. As shown in Figure 5, the BpCUC2-GFP (green fluorescent protein) fusion protein was localized to the nucleus.

**Figure 6:**
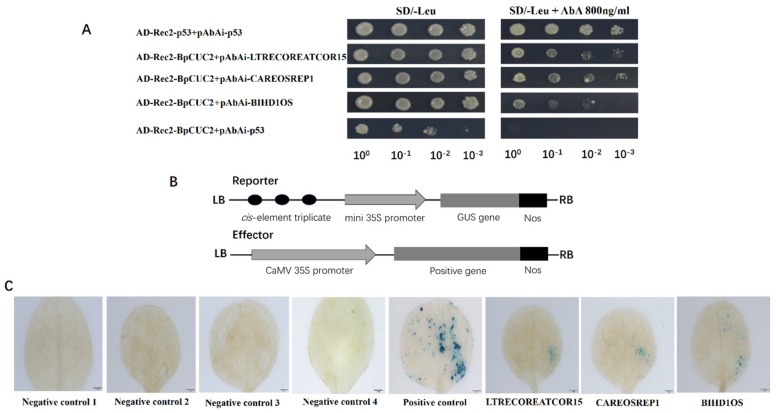
Analyses of BpCUC2 binding motifs. (**A**) Y1H analysis of binding BpCUC2 to LTRECOREATCOR15, CAREOSREP1, and BIHD1OS. (**B**) Structural diagram of the effector and reporter vector used in transient transformation assays. (**C**) GUS staining of the binding of BpCUC2 to the LTRECOREATCOR15, CAREOSREP1, and BIHD1OS motifs in tobacco leaves. Negative control 1–4: Tobacco leaves transformed reporter vectors with LTRECOREATCOR15, CAREOSREP1, and BIHD1OS motifs without BpCUC2 and co-transformed pCAMBIA1300-BpCUC2 and the reporter vector without motif sequences, respectively. Positive control: pCAMBIA1301. Scale bars = 1 mm.

To further investigate BpCUC2 targeted genes, we used the Transcription factor-centered Yeast One Hybrid [18] to identity BpCUC2 binding motifs. Using this system, a random short DNA sequence insertion library was generated as the prey DNA sequences and the BpCUC2 protein was used as the bait. After screening, we obtained about 15 positive clones of which five positive clones randomly sequenced. Following the analysis of insertion sequences on the PLACE (Plant Cis-acting Regulatory DNA Elements) database, we isolated several motifs that were recognized by BpCUC2. As shown in Table 1, “CCAGGCGTCGGG” (underlined bases represent part of the pHIS2 vector sequence) includes the CGACGOSAMY3 element “CGACG” [19] and LTRECOREATCOR15 element “CCGAC” [19]. “CAACTCCGAGTG” includes the CAREOSREP1 motif “CAACTC” [20], and “CTGCTTGTCAGG” contains the BIHD1OS motif “TGTCA” [20] and WRKY71OS motif “TGAC” [21]. We used standard yeast one hybrid (Y1H) assays to validate the binding of BpCUC2 to these motifs. Some of the motifs had transcriptional auto-activation activity and cannot be analyzed by yeast one-hybrid assays. Lastly, we identified LTRECOREATCOR15 (CCGAC), CAREOSREP1 (CAACTC), and BIHD1OS (TGTCA) that may be recognized by BpCUC2 using yeast one-hybrid assays. CAREOSREP1 has been identified as a GA-responsive sequence [20] and the LTRECOREATCOR15 plays a role in low temperature responsiveness [22]. BIHD1OS is the typical cis-element of the homeo-domain transcriptional factors [23]. The yeast one hybrid (Y1H) results indicated that yeast cells harboring the combination of AD-Rec2-BpCUC2 (effector) and reporters grew well on SD/-Leu/AbA selective medium, which demonstrate that BpCUC2 binds to these three elements (Figure 6A). To further verify the Y1H data, pCAMBIA1300-BpCUC2 constructs (effector) and vectors of three tandem copies of LTRECOREATCOR15, CAREOSREP1, and BIHD1OS fused with modified pCAMBIA1301-GUS (reporter), respectively, were co-transformed into tobacco leaves to drive GUS (β-glucuronidase) gene expression (Figure 6B). GUS expression was observed by staining the tobacco leaves after co-transformation with pCAMBIA1300-BpCUC2 using these motifs. No GUS staining was detected in co-transformed lines harboring pCAMBIA1300-BpCUC2 and the reporter vector without motif sequences and harboring reporter vectors with LTRECOREATCOR15, CAREOSREP1, and BIHD1OS motifs without BpCUC2 (Figure 6C). These data indicate that BpCUC2 may bind to the motifs of LTRECOREATCOR15 (CCGAC), CAREOSREP1 (CAACTC), and BIHD1OS (TGTCA).

We used RNA-sequences to identify differentially expressed genes (DEGs) in the *BpCUC2* OE lines. The apical buds of three-year old WT and three transgenic lines were collected. We generated four biological replicates for WT and each *BpCUC2* OE line, respectively. A total of 16 RNA-sequence libraries were constructed and sequenced. After sequence trimming, we achieved 491.6 million clean reads (28.7–31.8 million per library, Q30 ≥ 93%). Approximately 27.6–30.7 million clean reads per library could be mapped to the *Betula pendula subsp. pendula* genome (See Table S1). To further validate the DEG results, the expression of 12 randomly selected DEGs were verified by quantitative real-time reverse transcription PCR (qRT-PCR) analysis. Most qRT-PCR results were consistent with the DEG data (see Figure S7), which demonstrated the reliability of DEG results. Compared to WT, we identified a total of 688 DEGs in *BpCUC2* OE lines, including 533 upregulated genes and 155 downregulated genes. In the promoter regions of these DEGs, 275 contained LTRECOREATCOR15 motifs, 245 contained CAREOSREP1 motifs, and 592 contained BIHD1OS motifs.

In *Arabidopsis*, *CUC2* plays a significant role in auxin distribution [24]. In this study, we identified a range of auxin-related genes that were differentially expressed in *BpCUC2* OE lines. For example, the auxin biosynthesis-related genes *BpYUCCA5*, many *small auxin up RNA* (*SAUR*) family related genes (*BpSAUR21-like1*, *BpSAUR21-like2*, *BpSAUR21-like3*, *BpSAUR21-like4*, *BpSAUR24-like1*, *BpSAUR24-like2*, and *BpSAUR24-like3*) and IAA-amino acid hydrolase ILR1(*ILR1*) were up-regulated in *BpCUC2* OE lines. *BpARF2* was significantly down-regulated. We found that *BpLSH10-1* and *BpLSH10-2* are in the DEGs, which are two genes that belong to the same family of *CUC1* target genes *LIGHT-DEPENDENT SHORT HYPOCOTYLS 4*(*LSH4*) and *LSH3* [17]. *LATERAL SUPPRESSOR* (*LAS*/*SCL18*) are previously shown as direct *AtCUC2* target genes in *Arabidopsis thaliana*, and also in the DEGs [25] (See Table S2).

Plant growth, cell size, and cell number were inhibited in transgenic birch. Additionally, cell division and plant growth were interconnected [26]. Cyclin is a regulatory protein that interacts with cyclin-dependent kinases (CDKs) to control cell cycle progression. In addition to auxin related genes, we found that the cyclin-related genes *BpCyc U2-2 like* (Alternative name: *CYCP3;2 like*) were significantly up-regulated in *BpCUC2* OE lines. Cyclin-dependent protein kinase inhibitor *BpSMR6*, *BpCyclin-A3-1*, and *BpCyc-U4-1 like* were down-regulated in *BpCUC2* OE lines (See Table S2).

**Figure 7:**
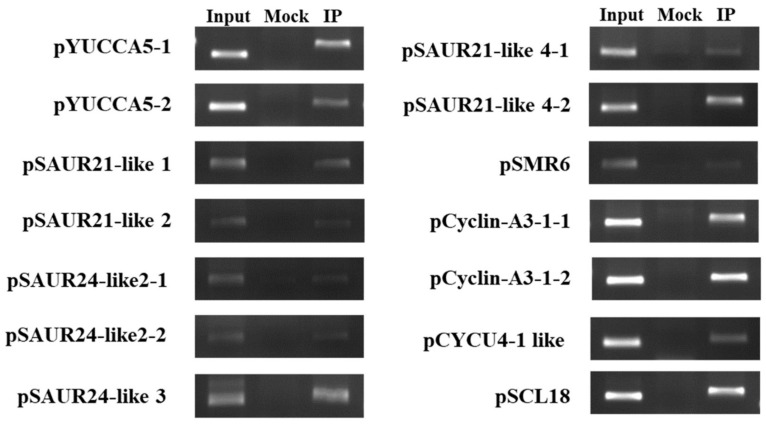
ChIP-PCR analyses of BpCUC2 target genes. Input: sonicated chromatin was used as a positive control. Mock: sonicated chromatin was immunoprecipitated with anti-IgG antibodies. IP: Sonicated chromatin was immunoprecipitated with anti-GFP antibodies. Experiments were performed in triplicate. Chromatin from buds was isolated from *BpCUC2* OE1 lines.

Since all *BpCUC2* OE lines expressed a BpCUC2-GFP fusion gene, we used GFP (green fluorescent protein) antibodies to perform ChIP-PCR and analyze the association of BpCUC2 with auxin and cyclin related genes via promoter binding. Since BpCUC2 binds to LTRECOREATCOR15, CAREOSREP1, and BIHD1OS, we selected genes whose promoters contain motifs for the ChIP-PCR experiments. Primers were designed around these motifs. The ChIP-PCR results indicated that BpCUC2 bound to the promoters of these genes, which suggests that it directly regulates a series of IAA-related and cyclin-related genes to mediate cell elongation and division in birch (Figure 7).

### 2.5. BpmiR164 Targets BpCUC2

**Figure 8:**
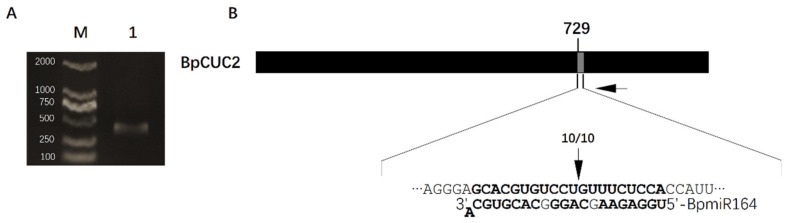
5′ RACE. (**A**) 2% agarose gel showing the amplified product of 5′ RACE on the *BpCUC2* transcript. M: DL2000 marker. lane 1: 5′ RACE product. (**B**) Scheme of target mRNA cleavage sites. Black box represents mRNA of BpCUC2. Small gray box represents putative cleavage sites. Complementary sequence of miRNA and target mRNA are displayed in the extended region. Numbers of sequenced 5′RACE clones corresponding to the site are indicated by vertical arrowheads. Horizontal black arrowheads represent 5′ RACE gene-specific primer sites.

Previous studies have shown that *miR164* targets the transcripts of *CUC2* [6]. The regulation of *CUC2* by *miR164* controls axillary meristem formation and leaf margin serration in *Arabidopsis* [8,27]. We identified two mature *miRNA164* sequences (*BpmiR164-1* and *BpmiR164-2*) in the *B. pendula* genome. To further confirm the regulatory relationship between *BpmiR164* and *BpCUC2*, 5′RACE was used to identify the cleavage sites of *BpCUC2*. The results showed that the 5′ ends of miRNA-guided cleavage products terminated in the middle of the *BpmiR164* and its complementary sequence (Figure 8), which was similar to *Arabidopsis thaliana* [27]. This indicates that *BpCUC2* is also a target of *BpmiR164* in *B. pendula*.

**Figure 9:**
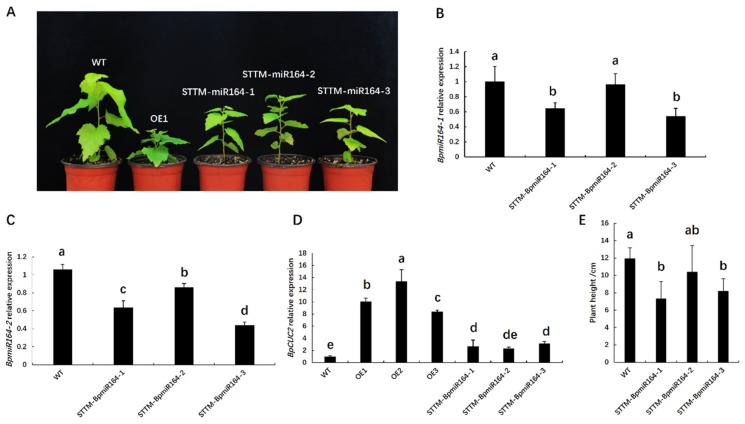
Regeneration and molecular analysis of STTM-BpmiR164 transgenic lines. (**A**) Phenotypes of STTM-BpmiR164 plants. (**B**,**C**) Relative expression of *BpmiR164-1* and *BpmiR164-2* in STTM-BpmiR164 transgenic plants. (**D**) Expression of *BpCUC2* in *BpCUC2* OE and STTM-BpmiR164 transgenic plants. Experiments were repeated on three occasions under identical conditions. (**E**) Plant height of four-month-old WT and STTM-BpmiR164 lines. A total of 15 plants were measured for each genotype. Different letters marked on each column represent significant differences when analyzed by one-way ANOVA and a multiple comparison using Duncan’s test at *P* < 0.05. Error bars represent the standard deviation (SD) of each line.

To further investigate the regulation of *BpmiR164* to *BpCUC2*, we generated three BpmiR164-repressing lines (STTM-BpmiR164-1, STTM-BpmiR164-2, and STTM-BpmiR164-3) using short tandem target mimic (STTM) technology via *Agrobacterium-mediated* transformation (see Figure S8) [28]. Since STTM can simultaneously target specific endogenous miRNAs for degradation, we constructed an STTM vector repressing *BpmiR164-1* and *BpmiR164-2*. However, the STTM-BpMIR164 lines were shorter than the wild type, though not as short as the *BpCUC2* OE lines (Figure 9A,E). We next examined the expression of *BpmiR164s* and *BpCUC2* in STTM-BpmiR164 lines. qRT-PCR indicated that the two *BpmiR164s* (*BpmiR164-1*, *BpmiR164-2*) were down-regulated in the STTM-BpmiR164 lines, while the expression of *BpCUC2* was up-regulated (Figure 9B–D), which indicates that *BpmiR164* negatively regulates *BpCUC2*. However, *BpCUC2* was modestly up-regulated in STTM-BpmiR164 lines. In the three STTM-BpmiR164 lines, *BpCUC2* was up-regulated 2.7, 2.3, and 3.1-fold, respectively. However, in *BpCUC2* OE lines, BpCUC2 was up-regulated 8 to 13-fold.

## 3. Discussion

*CUC2* regulates plant growth and development [15,29,30,31]. Previous studies have shown that *BpCUC2* with a typical NAC domain has a similar expression profile to *AtCUC2* in *Arabidopsis thaliana* [32], which suggests its involvement in the growth and morphogenesis of the *B. pendula*. Using a transgenic approach, we found that *BpCUC2* had a significant effect on many aspects of growth and development in *B. pendula*. Transgenic birch overexpressing *BpCUC2* exhibited bent and twisted apical stems, fusion of the petiole and stem, dwarfism, shortened internodes, and smooth leaf margins, which indicated that *BpCUC2* affected internode and leaf development in *B. pendula*. Similarly, *Arabidopsis* expressed a *miR164-resistant CUC2* gene showing an abnormal phyllotactic pattern [9]. In our previous studies, we transferred the *BpCUC2* gene into *Arabidopsis*, and the transgenic *Arabidopsis* showed a dwarf phenotype (unpublished), but the leaf shape did not differ from a wild type. In addition, *Arabidopsis* expressing 2x35S::CUC2 transgenic lines showed a mild growth reduction and wrinkled leaves, which differed from the severe growth reduction and smooth margins in *B. pendula* that overexpressed *BpCUC2* [7]. These results suggested that the regulatory mechanism of *CUC2* differs between *Arabidopsis* and *B. pendula*. This may be due to differences between herbaceous and woody plants, in which the regulation of *CUC2* in woody plants is more diverse. In summary, the over-expression of *BpCUC2* indicates that *BpCUC2* participate in normal internode and leaf development. However, it has been shown that the use of antigenic tags may lead to inappropriate conclusions, and that different tags have different effects on the functions of plant proteins [33]. *BpCUC2* OE lines use GFP-fusions that may affect *BpCUC2*′s functionality. For this reason, to study the native function of *BpCUC2*, untagged BpCUC2 overexpression lines should be produced in the future.

*Arabidopsis miR164* targets *CUC2* and regulates an array of growth and developmental processes [5,7,27]. In this case, we showed that *BpCUC2* is also a target of *BpmiR164* in *B. pendula*, and a cleavage site exists between the 11th and 12th positions of the complementary sequence, which is consistent with the cleavage site of *AtCUC2* in *Arabidopsis thaliana* by *AtmiR164* [34]. To determine the regulatory relationship between *BpmiR164* and *BpCUC2*, which may further functionally characterize *BpCUC2*, we regenerated three BpmiR164-repressed lines (STTM-BpmiR164-1, STTM-BpmiR164-2, and STTM-BpmiR164-3). Although the relationship between the miRNA and its target gene has traditionally been examined in miRNA overexpression studies or modified miRNA targets, recent evidence involving miRNA silencing through STTM revealed a regulatory relationship. In this study, *BpCUC2* expression was up-regulated in the three BpmiR164-repressed lines compared to WT controls. These results confirmed that *BpCUC2* expression is up-regulated by inhibiting *BpmiR164* expression (i.e., *BpmiR164* negatively regulates *BpCUC2* expression). However, the phenotype of STTM-BpmiR164 transgenic lines differed from that of the *BpCUC2*-overexpressed lines. We speculated that the degree of increase in *BpCUC2* expression in the BpmiR164-repressed lines was insufficient to produce clear phenotypic changes. Moreover, the results suggested that the phenotype was extremely sensitive to *BpCUC2* levels. Previous studies confirmed that STTM decreases miRNA abundance, but does not completely eliminate miRNA function [35]. In this study, the effects of *BpmiR164* silencing were relatively weak in the STTM-BpmiR164 lines, since the increase in mean relative *BpCUC2* expression levels in the STTM-BpmiR164 transgenic lines was 22.3% of those in BpCUC2-overexpressed lines. However, *miR164* expression overlaps with that of its target in *Arabidopsis* leaves and the vegetative shoot apex [8,27]. Yet, studies of expression pattern of *BpmiR164* and *BpCUC2* in tree species remain limited. Thus, the repression of *BpmiR164* may not lead to the defects observed following *BpCUC2* overexpression. Thus, the tight regulation of *BpCUC2* and *BpmiR164* expression require more thorough analysis in future studies.

Current research suggests that auxin biosynthesis and intercellular auxin transport in the meristem determine the polar localization and elongation rates of root cells [36]. As *BpCUC2* OE lines show inhibited epidermis cell elongation, we suspected that the overexpression of *BpCUC2* influenced IAA-related gene expression. Our transcriptome analysis revealed that the expression of several IAA-related genes [e.g., *SAURs* (the early auxin-responsive genes), *BpYUCCA5* (auxin biosynthesis-related gene), *ILR1* (IAA-amino acid hydrolase) and others], were significantly affected by *BpCUC2* overexpression. Global transcript profiling analysis revealed that auxin regulated various cyclin genes, such as *CYCB1* and *CYCA2* [37,38]. Additionally, the cell size and cell number decreased in the *BpCUC2* overexpressed lines, which were also associated with cyclin gene expression [26]. Thus, we examined cyclin-encoding genes among the DEGs in the *BpCUC2* overexpressed lines [e.g., *CDKs* (cyclin-dependent kinases), *BpCyclin-A3-1*, *BpCyc-U4-1*, and others]. We speculated these may be target genes of *BpCUC2*. In addition, BpCUC2 was found to bind on loci that are either up regulated or down regulated in *BpCUC2* OE lines, which indicates that BpCUC2 may act as both an activator and a repressor of transcription.

Transcription factor-centered Y1H and standard Y1H assays indicated that BpCUC2 can bind to the following three elements: LTRECOREATCOR15 (CCGAC), CAREOSREP1 (CAACTC), and BIHD1OS (TGTCA). Moreover, on the basis of our ChIP-PCR data, we determined that BpCUC2 can bind to the promoters of IAA-related and cyclin-related genes. Therefore, we hypothesized that BpCUC2 affects leaf shape and internode development by directly regulating the expression of a series of auxin-related and cyclin-related genes.

**Figure 10:**
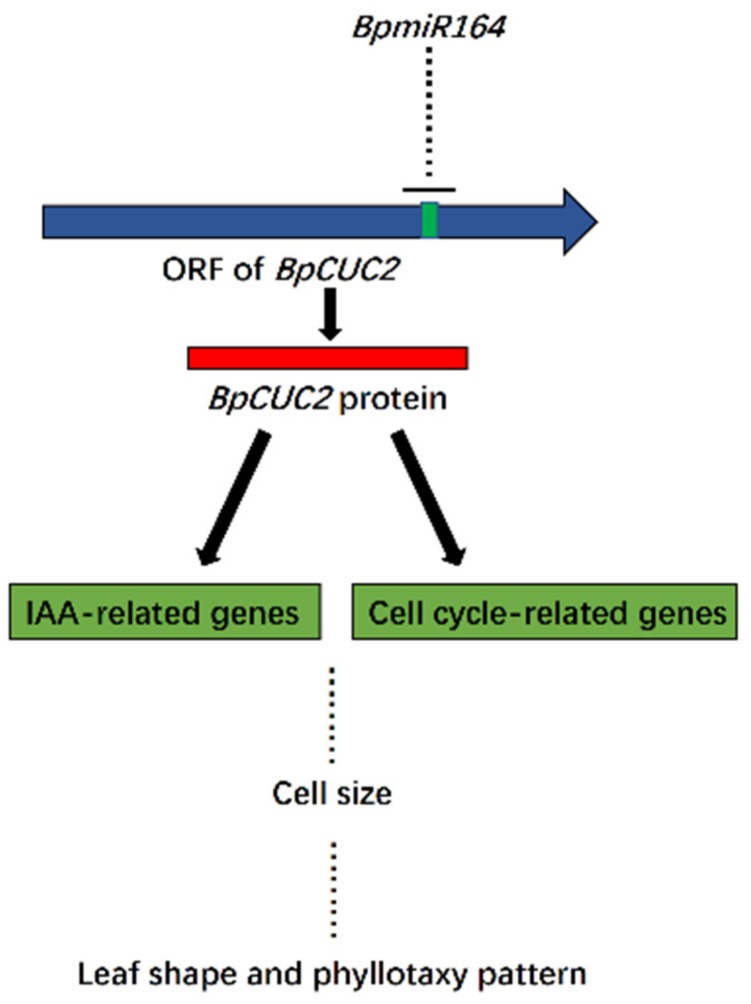
Schematic of BpCUC2 transcription factor involvement in regulatory networks. Arrows and arrowheads indicate regulatory effects. Lines with terminal bars indicate a repressive effect. Dotted lines indicate an unknown regulatory relationship.

Previous studies identified three upstream transcription factors that bind to the auxin response element (CACATG) of the *BpCUC2* promoter [32]. The current study may be useful for elucidating the function and regulation of *BpCUC2*. The data presented herein expand our knowledge of the function and regulatory network of BpCUC2 transcription factors in *B. pendula* (Figure 10).

Taken together, *CUC2* and the interacting *miR164* regulate various plant growth and development processes, including leaf margin dissections, lateral organ enlargement, and cotyledon and floral organ fusions. In this study, we identified and cloned *BpCUC2 in Betula pendula*, which was targeted by *BpmiR164*. Our results demonstrated that plants overexpressing *BpCUC2* produce smooth leaf margins and reduced stem cell size and cell number, which leads to shorter internodes. The downstream regulators were identified by transcriptome analysis and ChIP-PCR data. We hypothesize that BpCUC2 directly regulates a series of auxin-related and cyclin-related genes to influence leaf shape and the internode development in birch trees. Our results may provide meaningful clues on the BpCUC2 regulatory network and provide alternative materials and theoretical support for the future breeding of birch varieties.

## 4. Materials and Methods

### 4.1. Plant Growth Conditions

Mature seed embryos of wild type *B. pendula* were used as a transgenic explant, and the seeds were collected from a superior tree of free pollination in the birch national key forest seed base of Northeast Forestry University. Dry seeds were picked while bracts and fruit stems were removed. Samples were divided into small portions. Seeds were placed in plastic bags, sealed, and placed at −20 °C for storage.

Tissue culture seedlings of wild type and transgenic birch were grown in a tissue culture chamber at 25 °C ± 2 °C, 50% to 65% humidity, a photoperiod of 16/8 h, and an illumination intensity of 100–150 μmol·m^−2^·s^−1^. The multiplication and propagation of transgenic birch were performed as previously described [39]. The tissue culture seedlings of wild type and transgenic lines were cultivated on solid agar medium with woody plant medium (WPM) supplemented with 0.8 mg/L 6-BA, 0.02mg/L NAA, and 0.5mg/L GA_3_ in the tissue culture flasks. When the adventitious buds grew up, they were cut and cultivated on the solid agar medium with WPM (woody plant medium) supplemented with 0.2 mg/L NAA. After the seedlings grew up, they were transplanted to the birch national key forest seed base of Northeast Forestry University. Wild type birch (WT), *BpCUC2* overexpressing lines (OE1-OE3), and *BpmiR164* inhibiting expression lines (STTM-BpmiR164-1, STTM-BpmiR164-2, and STTM-BpmiR164-3) were transplanted into 30 plants. In early June, the tissue culture seedlings were transplanted into nursery cups. In the early spring, 30 seedlings with the same conditions were selected and placed in 21 cm × 21 cm pots. The seedling growth substrate was grass charcoal: river sand: black soil (*v*/*v*) = 4:2:2. Identical amounts of substrate were added to each pot, plastic trays were placed beneath, and the seedlings were placed in a plastic greenhouse for routine management. Plant positions were varied during growth to avoid the influence of environmental factors.

### 4.2. Identification and Bioinformatics Analysis of BpCUC2

We aligned the protein sequences of *B. pendula* to other species using BLAST software (https://blast.ncbi.nlm.nih.gov/Blast.cgi). The top hits of *AtCUC2* in *B. pendula* were identified as *BpCUC2* genes (No. Bpev01.c0346.g0003.mRNA1, see Table S5). The NAC family were collected from the literature and GenBank (GenBank [http://www.ncbi.nlm.nih.gov/]). Gene names and GenBank accession numbers are listed in Table S3.

Multiple sequence alignments were generated using CLUSTAL X (version 1.81) with default parameters [40], and phylogenetic analysis was performed using the neighbor-joining method [41]. *BpCUC2* annotations were added to the NCBI Conserved Domain Database [42].

### 4.3. Vector Construction and Birch Transformation

To overexpress *BpCUC2*, full-length cDNA of *BpCUC2* was isolated from birch leaves by PCR and cloned into the pCAMBIA1300-GFP vector using the cauliflower mosaic virus 35S (CaMV 35S) promoter. To silence *BpmiR164* expression, short tandem target mimic (STTM) approaches were used as described [43]. The STTM-BpmiR164 structure was used to capture *BpmiR164* without its cleavage (5′ catttggagaggacagcccAAGCTTTGGAGAAGCAGctaGGCACGTGCAGttgttgttgttatggtctaatttaaat

atggtctaaagaagaagaatAGCATGTGCCCctaTGCTTCTCCAGAATTCggtacgctgaaatcaccag 3′). Underlined nucleotides highlight the sequences complementary to *BpmiR164s* with tri-nucleotide mismatches in the middle.

The 35S::BpCUC2 and STTM-BpmiR164 constructs were delivered into the *Agrobacterium tumfaciens* strain EHA105 via electroporation [38] and the birch genome using *Agrobacterium-mediated* transformation [32]. The wild type *B. pendula* seeds were immersed in water for two to three days until the seeds are swollen and used as the transgenic explants. After the seeds were sterilized in 30% hydrogen peroxide for 15 min, they were rinsed with sterile water 2~3 times for use. *Agrobacterium tumfaciens* containing the 35S::BpCUC2 and STTM-BpmiR164 constructs was used to infect the longitudinally cut seeds. Afterward, the explants were placed onto co-cultivation medium in the dark for 2 days and then planted on selective medium. After 20 days, resistant explant shoots were placed on differential medium. Growing shoots were placed onto rooting medium. The culture medium was as previously described [39]. The selection media consisted of WPM with 0.8 mg/L BA, 0.02 mg/L NAA, 0.5 mg/L GA_3_, 50 mg/L hygromycin, and 200 mg/L cefotaxime. 

### 4.4. Analysis of Transformants

CUC2-F (5′-ATGGAGTATTCGTACAACTATTTTG-3′) and CUC2-R (5′-GAAGGTCCACATGCAGTCAAG-3′) primer pairs were used for *BpCUC2* OE (35S::BpCUC2 transgenic plants) PCR validations. STTM-common-real-PF (5′-CATTTGGAGAGGACAGCCCAAG-3′) and STTM-common-real-PR (5′-CTGGTGATTTCAGCGTACCGAA-3′) primer pairs were used for STTM-BpmiR164 transgenic plant PCR validations.

For the analysis of *BpCUC2* expression, total RNA was extracted from the buds of wild type (WT) and *BpCUC2* OE lines using Universal Plant Total RNA Extraction Kits (BioTeKe). Quantitative real-time PCRs were performed using quantitative SYBR green PCR Master Mix (Toyobo Co., Ltd., Osaka, Japan) and an ABI 7500 Real-Time PCR system. Amplifications were performed as follows: 45 cycles at 95 °C for 30 s, 95 °C for 15 s, and 58 °C for 40 s. The *18S* rRNA was used as an internal control. Gene-specific primers (i.e., *BpCUC2*-F/ *BpCUC2*-R) and internal reference primers (i.e., *18S*-F/*18S*-R) (see Table S4) were designed using the Primer-BLAST tool in NCBI (https://www.ncbi.nlm.nih.gov/tools/primer-blast/). Data were analyzed using the 2^−ΔΔC*T*^ method [44].

For *BpmiR164* expression analysis, total RNA was extracted from the buds of WT and STTM-BpmiR164 transgenic plants using the CTAB (Hexadecyltrimethy Ammonium Bromide) method [45]. miRNA First-Strand cDNA synthesis was performed via TransScript ^®^ miRNA First-Strand cDNA Synthesis SuperMix (TransGen Co., Ltd., Beijing, China). Quantitative real-time PCRs were performed using TranScript Green miRNA Two-Step qRT-PCR SuperMix (TransGen Co., Ltd., Beijing, China). Amplifications were performed as follows: 94 °C for 30 s, 45 cycles of 94 °C for 5 s, and 60 °C for 34 s. *U6* was used as an internal control. Gene-specific primers (i.e., *BpmiR164*-1, *BpmiR164-2*) and internal reference primer (i.e., *U6*) sequences are listed in Table S4. Data were analyzed using the 2^−ΔΔC*T*^ method [44].

### 4.5. Analysis of BpCUC2 Expression

To analyze the expression pattern of *BpCUC2*, total RNA was extracted from the buds of seedling stage and one-year old WT and *BpCUC2* OE lines as well as the leaves from two-year old WT and *BpCUC2* OE lines at different developmental stages (May to August) using Universal Plant Total RNA Extraction Kits (BioTeKe). Quantitative real-time PCRs were performed using a quantitative SYBR green PCR Master Mix (Toyobo Co., Ltd., Osaka, Japan) and an ABI 7500 Real-Time PCR system. Amplifications were performed as follows: 45 cycles at 95 °C for 30 s, 95 °C for 15 s, and 58 °C for 40 s. The 18S rRNA was used as an internal control. Gene-specific primers (i.e., *BpCUC2-F*/*BpCUC2-R*) and internal reference primers (i.e., *18S-F*/*18S-R*) were shown in Table S4. Data were analyzed using the 2^–ΔΔC*T*^ method [44].

### 4.6. Internode Epidermal Cells Analysis

Since the length of epidermal cells in the same internode are uniform, the number of cells between each internode was calculated by dividing the internode length by the average length of the cells. Five plants were measured for each genotype.

### 4.7. Histological Analyses and Microscopy

The one-year-old WT and *BpCUC2* OE lines were transplanted into plastic greenhouses and the buds and stem regions were incubated in FAA solution at 4 °C for 48 h. After dehydration, tissues were cleared, infiltrated, and embedded in paraffin wax, as previously described [46]. Sections (10 µm) were stained with safranin O-fast green. An Olympus BX43 microscope was used for section analysis. A total of five plants were measured for each genotype.

### 4.8. Phenotypic Characterization

Plant heights were measured in one-year-old WT and *BpCUC2* OE lines and four-month-old WT and STTM-BpmiR164 lines. Internode lengths were assessed on the stems of one-year-old WT and *BpCUC2* OE lines. The top three internodes of the stems were not assessed since the elongation was incomplete. Each genotype measured 15 plants. For leaf shape analysis, the 1st~8th leaves of the main branch were removed from WT and *BpCUC2* OE lines. According to the Manual of Leaf Architecture, the basal angles of the 1st, 3rd, 5th, and 7th leaves and the numbers of orders of leaf teeth of the 1st to 8th leaves of WT and *BpCUC2* OE lines were measured [47]. The leaf base angle is the angle from the vertex to the points where a line perpendicular to the midvein at 0.25 lm from the base intersects the margin (lm = from the proximal to the distal midvein) [47]. As *B. pendula* leaves have three distinct sizes teeth, the largest is the 1st order teeth, the second is the 2nd order teeth, and the smallest is the 3rd order teeth. Analysis of early leaf development in tissue culture seedling of WT and *BpCUC2* OE line was performed by directly dissecting the buds and peeling off the leaves layer by layer. Zeiss Lumar.V12 microscopy was used for image analysis. A total of five plants were measured for each genotype.

### 4.9. Subcellular Localization

The 35S:BpCUC2-GFP expression vector was transfected into onion epidermal cells using particle bombardment technology. After one day of incubation in the dark, onion tissues were imaged via confocal microscopy.

### 4.10. Transcriptome Analysis

Buds of three-year-old WT and *BpCUC2* OE lines was taken for transcriptome analysis. After mixing, samples were placed in liquid nitrogen and sent to the Annoroad Company (Annoroad Gene Technology Co., Ltd., Beijing, China). After RNA quality examination, library construction was performed. Libraries were sequenced on an Illumina platform (Illumina, San Diego, CA, USA). Using STAR (ver. 2.4.0) software [48], clean reads were mapped to *B. pendula* mRNA reference sequence (https://genomevolution.org/coge/GenomeInfo.pl?gid=35079). The function ′DESeq′ from the R package ′DESeq2′ was used with its default parameter to analyze the adjusted P value of each genes′ expression difference [49]. Twelve replicates of OEs were treated as a whole to derive a single fold change versus WT. DEGs were restricted and false discovery rates with *p* < 0.05 and fold change (FC) ≥2 were considered. We annotated the DEGs and their proteins through a comparison to NCBI (see Table S5). Data were submitted in the BioProject database under BioProject ID: PRJNA540156 (https://www.ncbi.nlm.nih.gov/bioproject/PRJNA540156).

To verify the accuracy of transcriptome, gene-specific primers (see Table S4) were designed using the Primer-BLAST tool in NCBI (https://www.ncbi.nlm.nih.gov/tools/primer-blast/). Quantitative real-time PCRs were performed as follows: 45 cycles of 95 °C for 30 s, 95 °C for 15 s, and 58 °C for 40 s. The *18S* rRNA was used as an internal control. Data were analyzed using the 2^–ΔΔC*T*^ method [44].

### 4.11. Binding of BpCUC2 to Motifs

TF-Centered Y1H was used to investigate the binding motifs of BpCUC2, as previously described [50]. The ORF (Open Reading Frame) of BpCUC2 was cloned into pGADT7-Rec2 (AD-Rec2-BpCUC2) using the infusion method following the user manual (In-Fusion ^®^ HD Cloning Kit). In the TF-Centered Y1H system, random short DNA sequences were cloned into pHIS2 to generate prey DNA sequence libraries with specific transcription factors as bait. pHIS2 plasmids were extracted from positive clones identified by TF-centered Y1H analysis and sequenced. The PLACE (http://www.dna.affrc.go.jp/PLACE/) database was used to analyze random DNA sequences for known motifs [51]. The random DNA sequences and motifs predicated by PLACE (Plant Cis-acting Regulatory DNA Elements) software is shown in Table 1.

Tobacco transient expression experiment was used to analyze the binding of BpCUC2 to different motifs. Three tandem copies of LTRECOREATCOR15, CAREOSREP1, and BIHD1OS were fused to the 35S CaMV minimal promoter (−46 bp to +1) to drive the GUS gene in a modified pCAMBIA1301 vector (in which the 35S:hygromycin region was deleted) [52]. Effector vectors were co-transformed into tobacco leaves with each reporter vector using *Agrobacterium tumefaciens*-mediated transient transformation [50].

### 4.12. ChIP Assay

The *BpCUC2* OE1 buds were used for ChIP assays, as previously described [53]. Sonicated chromatin was immunoprecipitated with GFP antibodies (Abmart) (IP), and chromatin was immunoprecipitated with IgG antibodies as negative controls (Mock). According to the transcriptome analysis, 20 genes were selected and primers were designed for the promoters of these genes (~1000 bp upstream of the initiation codon). Input, Mock, and ChIP samples were used as templates for ChIP-PCRs. Primer sequences used for ChIP amplifications are listed in Table S6.

### 4.13. 5′ RACE

5′RACE was performed using FirstChoice ^®^ RLM-RACE Kits (Invitrogen, CA). Total RNA was isolated from one-year-old *B. pendula* plants and directly ligated to the 5′ RACE Adapter (5′-GCUGAUGGCGAUGAAUGAACACUGCGUUUGCUGGCUUUGAUGAAA-3′) without further modifications. Random decamers were used to prime cDNA synthesis with reverse transcriptase. Primers were as follows: 5′ RACE Outer primer (5′-GCTGATGGCGATGAATGAACACTG-3), 5′ RACE gene-specific outer primer (5′-CACATGCAGTCAAGCTCAGTAGGA-3′), 5′ RACE Inner Primer (5′-CGCGGATCCGAACACTGCGTTTGCTGGCTTTGATG-3′), and 5′ RACE gene specific inner primer (5′-AAGGTTCTCCTGCAAGGACCTCA-3′). The 5′ RACE products were gel purified, cloned, and sequenced.

## 5. Conclusions

As a perennial tall tree, birch is the pioneer species of secondary forests in Northeastern China. It has a fast growth rate, strong cold resistance, excellent material properties, and high survival rate of artificial planting. It also has a wide range of applications in furniture manufacturing and landscaping. Therefore, research on the key genes regulating the growth and development of birch can provide a theoretical basis for forest molecular breeding and provide materials for obtaining excellent new varieties of forest trees. Our results showed that *BpCUC2* directly regulates a series of auxin-related and cyclin-related genes to influence leaf shape and the internode development in *Betula pendula*. Our results may provide meaningful clues on the *BpCUC2* regulatory network and the theoretical support for the future breeding of birch trees.

## Figures and Tables

**Table 1:** Random DNA sequences and motif predictions.

Clone Number	Random DNA Insertion Sequence (5′-3′) with Two Sides of the Flanking Sequences	Motif Prediction
3	CCAGGCGTCGGG	CGACGOSAMY3 (CGACG), LTRECOREATCOR15 (CCGAC)
1	CAACTCCGAGTG	CAREOSREP1 (CAACTC)
1	CTGCTTGTCAGG	BIHD1OS (TGTCA), WRKY71OS motif (TGAC)

## Supplementary Materials

Supplementary materials can be found at https://www.mdpi.com/1422-0067/20/19/4722/s1.
