# Supplementary material for: Overexpression of BpCUC2 Influences Leaf Shape and Internode Development in Betula pendula
Source: Int J Mol Sci. 2019 Sep 23;20(19):4722. doi: 10.3390/ijms20194722 (PMC6801603; doi:10.3390/ijms20194722)
Supplement: Supplementary file 1 [file ijms-20-04722-s001.zip › Supplementary FigureS1-S6.docx]

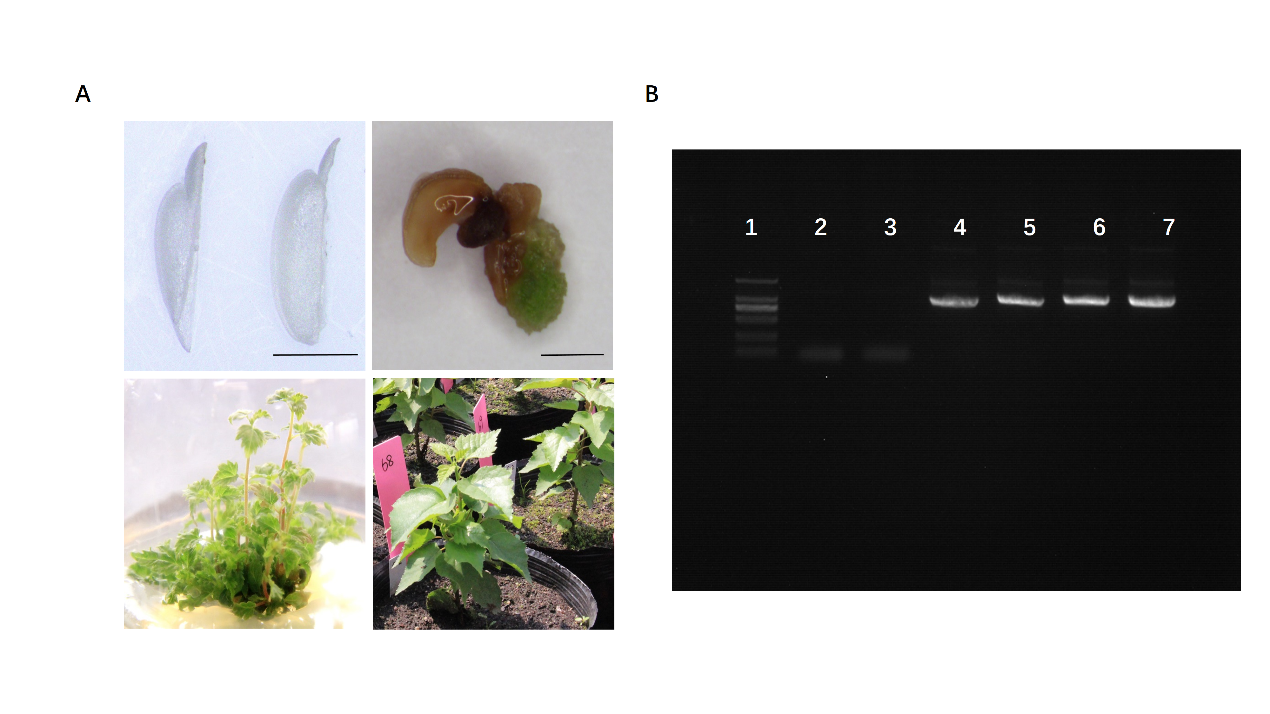


**Figure S1. Regeneration and molecular analysis of BpCUC2 OE lines.** (A) Regeneration of BpCUC2 OE lines. From left to right, from top to bottom are birch zygotic embryos, hygromycin-resistant buds, differentiation of hygromycin-resistant buds and transplanted plants. (B) PCR validations on OE lines; Lane1, DNA Marker DL2000; deionized water and WT gDNA were utilized as the PCR templates for Lanes 2–3; pCAMBIA1300-BpCUC2-GFP plasmid was utilized as the PCR templates for Lanes 4; OE1-OE3 gDNA were utilized as the PCR templates for Lanes 5–7. Scale bar: 1mm.


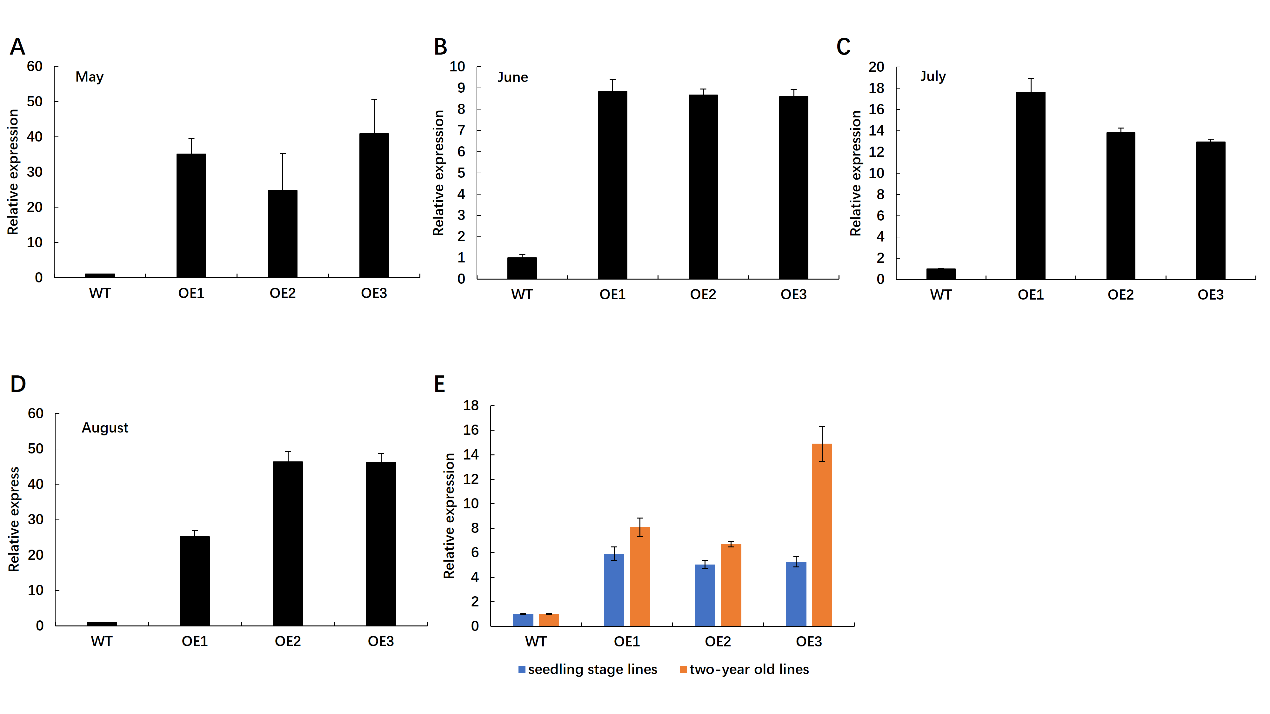


**Figure S2. Expression pattern analysis of of *BpCUC2*.** (A-D) *BpCUC2* relative expression in leaves of two-year old WT and transgenic lines in May, June, July and August, respectively. (E) *BpCUC2* relative expression in the buds of seedling stage and one-year old WT and transgenic lines. The experiments were replicated three times under identical conditions, error bars represent the standard deviation (SD) of each line.


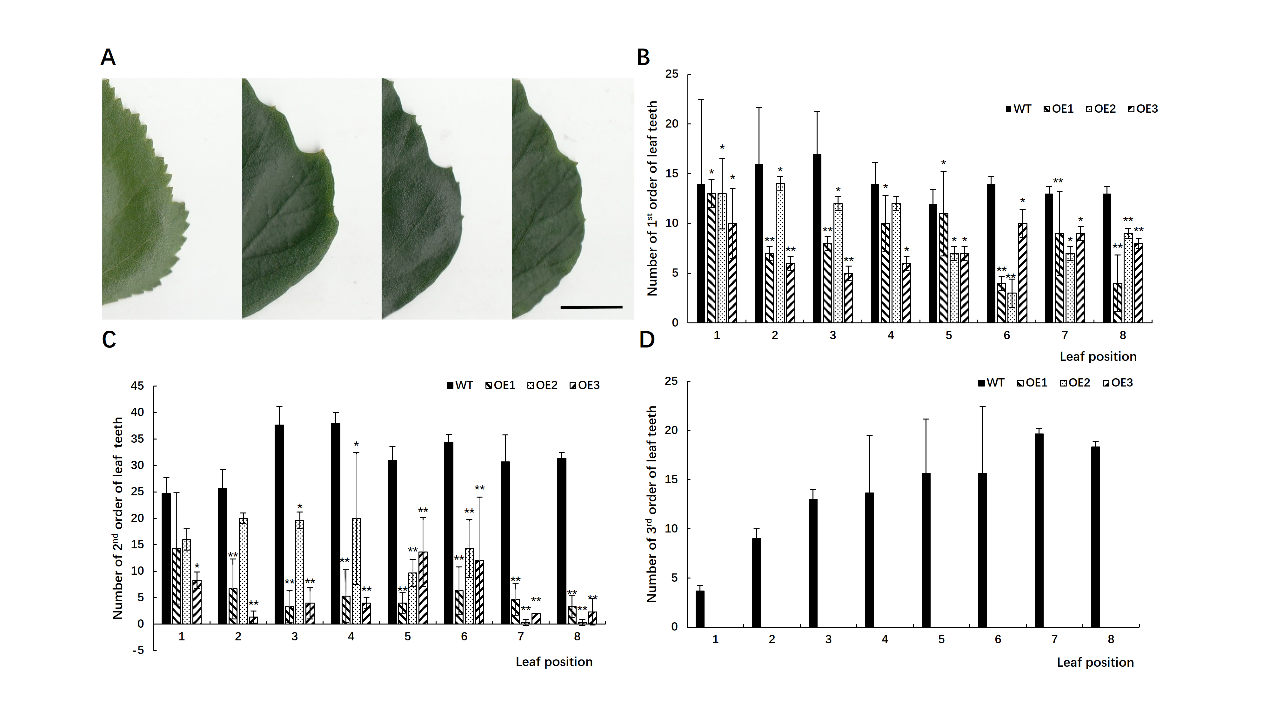


**Figure S3. Number of orders of teeth in WT and BpCUC2 OE lines.** (A) Leaf margin of WT and OE lines. (B), (C) and (D) Number of 1^st^, 2^nd^ and 3^rd^ order of leaf teeth. Two stars indicate a statistically significant difference between WT and BpCUC2 OE lines at P < 0.05 when analyzed by one-way ANOVA. A total of five plants were measured for each genotype. Scale bar: 1cm


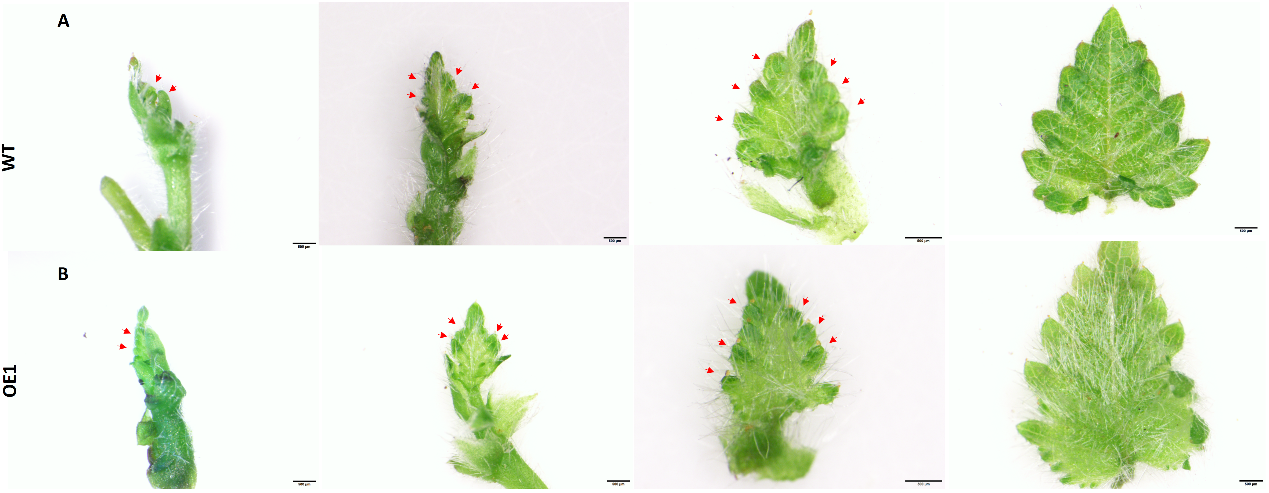


**Figure S4. Early leaf development in tissue culture seedling of wild type (A) and *BpCUC2* OE1 line (B).** Figures of each line, from left to right, represents the order of the leaves from inside to outside. Arrowheads point to leaf teeth. Scale bars: 500μm


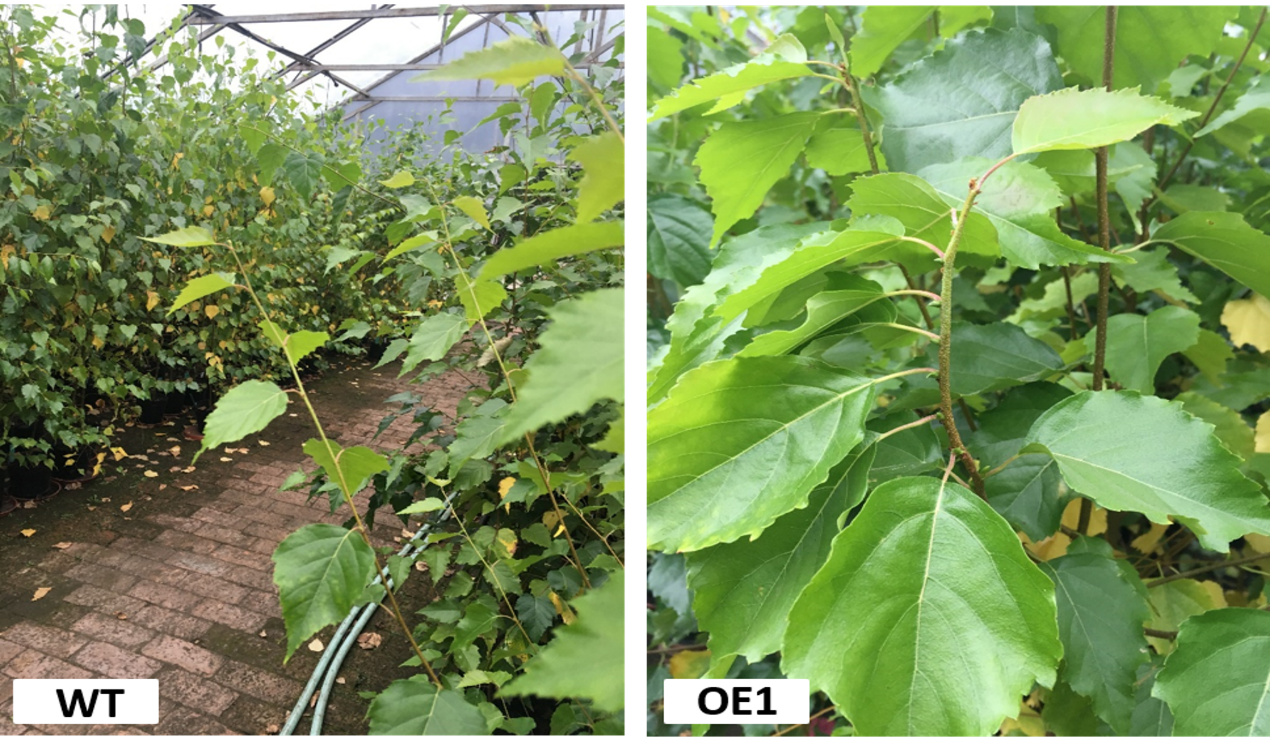


**Figure S5. Abnormal phyllotaxy of two-year old WT and *BpCUC2* OE1 line.**


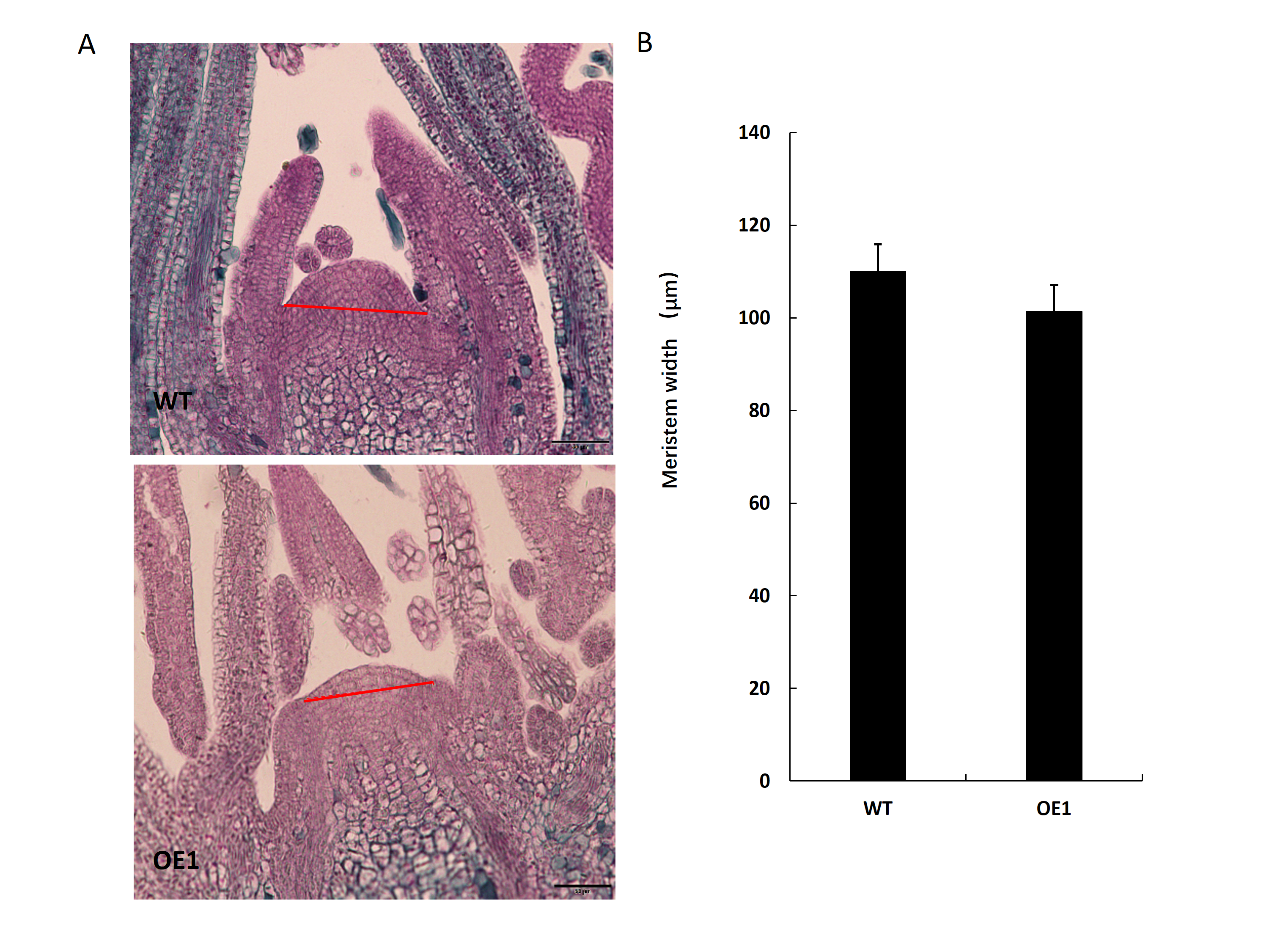


**Figure S6. Meristems width is unaltered between WT and BpCUC2 OE line.** (A) Meristem of WT and BpCUC2 OE line. (B) Meristem width of WT and BpCUC2 OE line. Standard deviations (bars) are indicated. Five plants were measured per genotype. A total of five plants were measured for each genotype. Scale bar: 50μm.


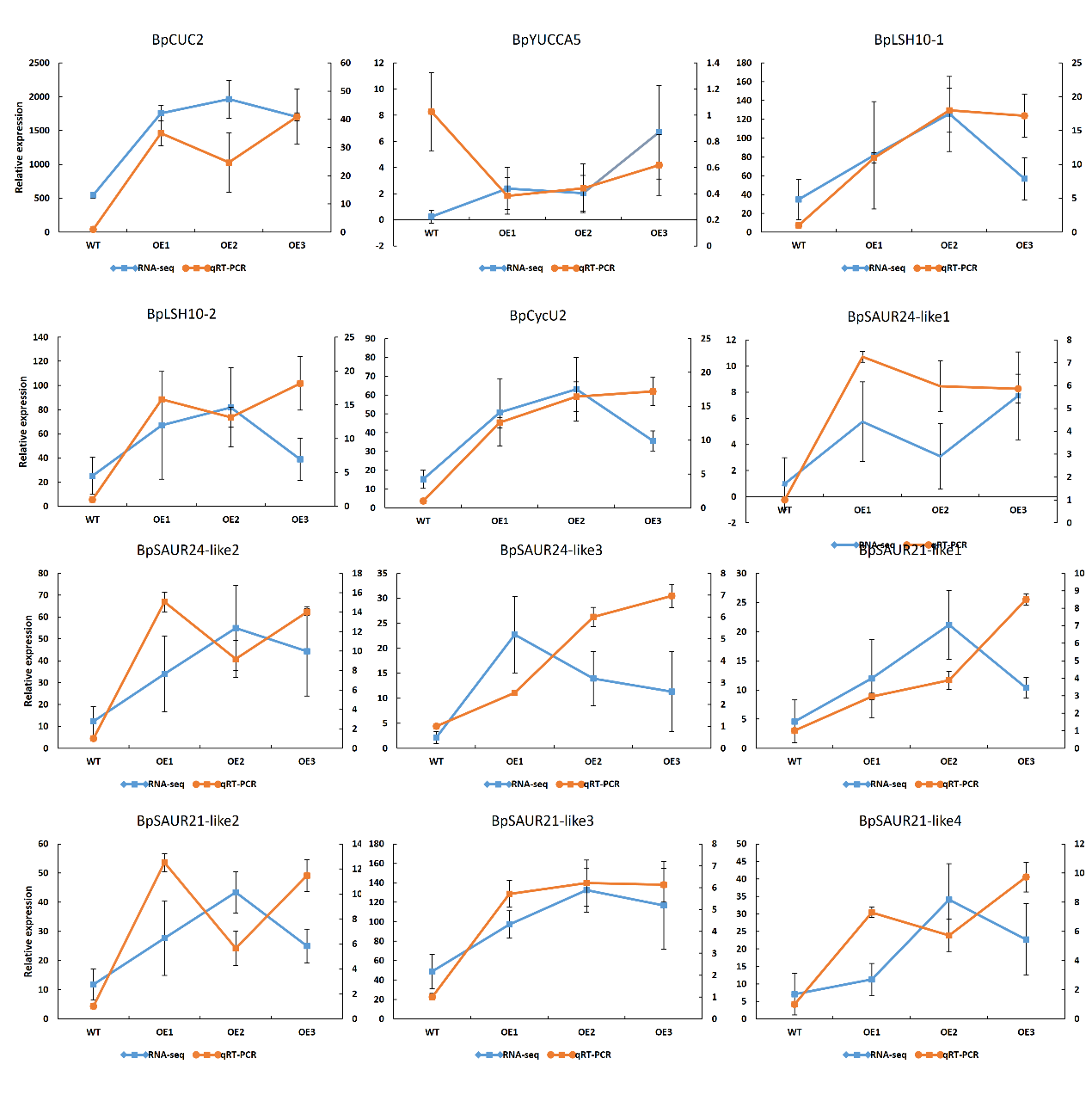


**Figure S7. Confirmation of the DGE results using qRT-PCR analysis.** Relative expression level by real-time quantitative PCR was calculated using 2^-ΔΔCT^ methord taking *18s* as internal calculation. Each experiment was repeated three times. The red line represented the result of qRT-PCR and the blue line represented the result of RNA-seq. Error bars were obtained from threereplicates of the qRT-PCR. The leaf Y axis represented the relative expression of RNA-seq result and the right Y axis represented the relative expression of qRT-PCR result.

**
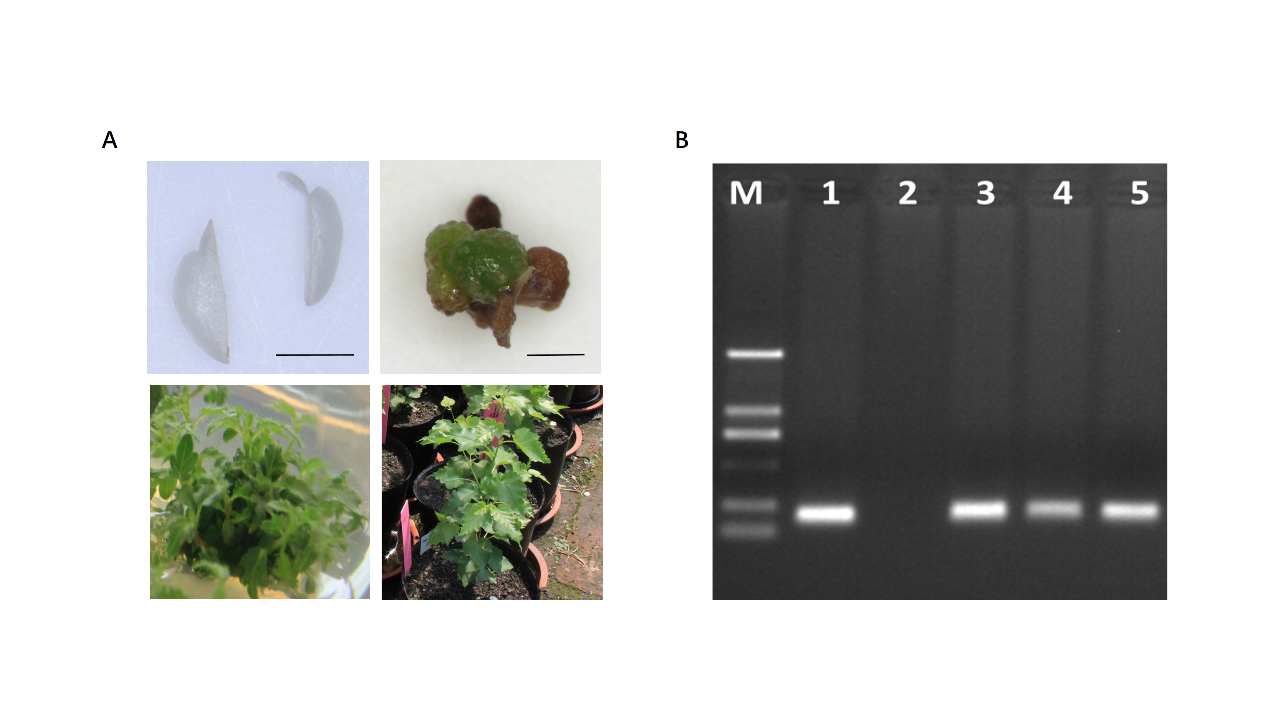
**

**Figure S8. Regeneration and molecular analysis of STTM-BpmiR164 transgenic lines.** (A) Regeneration of STTM-BpmiR164 transgenic lines. (B) PCR detection of transgenic lines. Marker: DL2000, Lane 1 template is STTM-miR164 plasmid, Lane 2 template is ddH2O, Lane 3-5, templates are the gDNA of STTM-BpmiR164-1, STTM-BpmiR164-2, STTM-BpmiR164-3. Scale bar: 1mm.
